# Preferences for woodland activities and forest features as predictors of well-being after forest visits: Evidence from a nationally representative survey in Slovakia

**DOI:** 10.1007/s13280-024-01982-0

**Published:** 2024-02-07

**Authors:** Jozef Výbošťok, Magdaléna Pichlerová, Kiki Ekiawan Lamatungga, Dhanalakshmi Tamatam, Dilek Önkal, Daniel Halaj, Viliam Pichler

**Affiliations:** 1 Faculty of Forestry, Technical University in Zvolen, T. G. Masaryka 24, 960 01 Zvolen, Slovakia; 2 Faculty of Ecology and Environmental Sciences, Technical University in Zvolen, T. G. Masaryka 24, 960 01 Zvolen, Slovakia; 3 Department of Marketing, Operations and Systems, Newcastle Business School, Northumbria University, Newcastle upon Tyne , NE1 8ST UK

**Keywords:** Environmental preferences, Forest smell, Forest visits, Restorative environment, Stress relief, Subjective well-being

## Abstract

The link between subjective well-being (SWB) and forest visits is increasingly driving the development and preservation of restorative forest environments in numerous countries. However, there is limited knowledge regarding the effect of people's preferences for forest patterns and activities on this connection. Here we investigated whether associations exist between the preferences for certain forest features and activities, and the SWB increase and stress reduction in response to forest visits. A nationwide digital survey was administered to a representative sample of the Slovak population. The recollection-based data obtained from one thousand respondents were analysed through agglomerative clustering and ordinal regression. The analyses revealed that improved SWB and stress reduction were associated with preferences for uneven-aged forests, forest smell, as well as recreational, but not provisioning forest activities. The respective interrelationships explained up to 20% of SWB increase and stress reduction after forest visits. The results suggest that recollection-based study findings can be generalized for real-world forests and that forest management can contribute to the well-being of forest visitors by shaping the diversity of woodlands and their sensory experiences.

## Introduction

A global survey conducted in Germany, Sweden, the USA, China, Brazil, Slovakia, and Italy revealed that people associate forests mostly with healthy environments and recreation (Consumers and Biobased Materials, 2018). During the COVID-19 pandemic, forests had become one of the few outdoor environments available to people, and in many countries, people made more forest visits than before (Beckmann-Wübbelt et al. 2021; da Schio et al. 2021; Pichlerová et al. 2021). Studies indicated that people felt deprived of social contact, work, and cultural and sports activities, and many people experienced a shift towards negative emotions as a result of the pandemic (Cerbara et al. 2020; Esterwood and Saeed 2020; Xiang et al. 2020). Even before the COVID-19 pandemic, mental health problems affected 1 in 6 people in Europe, including Slovakia, with the situation worsening during the pandemic (Amand-Eeckhout 2023). Corbett et al. (2023) reported persistent mental health decline in older adults (50 years and older) even after the COVID-19 pandemic. This trend has been partially countered by a global increase in nature experiences that showed a consistent positive association between nature exposure and improved mental health (Labib et al. 2022).

The explanation of the restorative capacity of natural environments and forests draws on attention restoration and stress reduction theories (ART and SRT, respectively). ART (Kaplan and Kaplan 1989) emphasizes the restorative environment’s capacity to produce a partly involuntary fascination. Alternatively, SRT postulates that natural environment triggers a shift in feelings towards a more positive emotional state linked with attention and subsequent conscious processing (Ulrich 1983; Ulrich et al. 1991). Nature-based outdoor activities, forest visits, and environmental simulation have been shown to facilitate restoration, stress reduction, cognitive function, physical and mental health, and subjective well-being (SWB) on both individual and public scales (Hartig et al. 1996; Kuo 2015; Lanki et al. 2017; Stigsdotter et al. 2017; de Brito et al. 2020; Gallis 2020). However, there is a need to study relationships between specific forest features, types of activity, SWB, and mental health to maximize the benefits of forest planning and public health policies within the forest ecosystem services framework (Milcu et al. 2013; Doimo et al. 2020; Meneguzzo and Zabini 2021; Piva et al. 2022; Clark et al. 2023). Thus far, considerable attention has been paid to the integration of popular provisioning activities, such as berry- or mushrooms-picking, into the rural economy or optimizing the infrastructure for various types of recreational activities typically performed in forests (Simpson et al. 2008; Riedl et al. 2020). However, it was suggested that the forest environment, rather than the activity performed in forests, could nurture absorption and intense positive moods as important components of SRT and ART and that significant positive effects of a profound experience with nature on people can persist over a long time (Williams and Harvey 2001; Mathers and Brymer 2022).

The perceptual properties of the natural environment experiences of its restorative potential, and one’s actions in—and interactions with—the environment shape the corresponding preferences (Beute and de Kort 2018). Recently, a discrete choice experiment with digitally calibrated forest images (*N* = 160) showed similar results for visitor preferences and restoration, mainly concerning canopy and trees density (Ebenberger and Arnberger 2019). However, the knowledge regarding interrelationship between SWB and preferences for forest activities and forest features remains very limited, e.g., with regard to representativeness, auditory and olfactory stimuli, forests with other than primary restorative or recreation functions and so on. Therefore, the primary research objective of this study was to reveal specific recollection-based associations between SWB and stress reduction after forest visits, and the preferences for certain forest activities, forest features, and forest-derived sensory stimuli experienced in Slovak forests, representing a considerable part of forest diversity within the Temperate Zone of Europe. It was anticipated that the results could inform public health and forestry policies in providing quality forest environments for mental restoration in Slovakia and other countries with comparable natural and societal conditions. The study had two working hypotheses: 1) Increased SWB and stress reduction are significantly associated with the respondents’ preferences for certain forest features and types of activity; 2) Provisioning forest activities, owing to their direct material or commercial benefits, affect SWB more than recreational activities.

## Materials and methods

A nationwide survey was conducted in Slovakia to investigate anticipated links between SWB and stress experienced during the early phase of the COVID-19 pandemic, and the forest visitor activities and preferences for certain forest features or sensory stimuli.

### Basic demographic characteristics

Slovakia is a country located in Central-Eastern Europe with an area of ca 49 thousand km^2^ and a population of approximately 5 million people, of which 54% live in cities (Dická et al. 2019). Approximately 30%, 66%, and 68% of the population lives within 1 km, 5 km, or 10 km, respectively, of nearby forests (Newton et al. 2020). As forests cover some 42% of the Slovak territory, it was assumed that they became one of the few environments available for outdoor recreation during the COVID-19 pandemic. According to Pichlerová et al. (2021, 2023), the number of forest visits per person per month increased by approximately 9%, especially in the 30–44 years age category.

### Forest cover

Slovak forests are mostly semi-natural and mixed. According to the required site conditions, their tree species composition comprises European beech (*Fagus sylvatica*), oaks (*Quercus rubra*, *Q*. *petraea*), Norway spruce (*Picea excelsa*), silver fir (*Abies alba*), and other indigenous species. While the country's forestry policy leans on the premise that sustainable forest production secures all the other forest ecosystem services, the forest law allots forests for three main purposes. The respective categories comprise commercial forests, protective forests with prevailingly environmental functions, and special purpose forests, e.g., urban forests with recreation function and forests in nature conservation areas (73%, 17%, and 10% of the forested areas, respectively). In commercial forests, forest structure is determined by the prevailing shelterwood-cut management resulting in even-age class forests interspersed with uniform cutting areas (mostly < 2 ha). In commercial forests, trees are usually cut at 100 years of age.

### Survey instrument and administration

The cross-sectional survey was conducted in the summer 2020 after a moderate relaxation of the pandemic measures and restrictions following the first pandemic wave. The survey was performed on a representative sample of respondents from a panel of individuals living in Slovakia. The required sample size was determined using the formula by Krejcie and Morgan (1970) for strata in which the population size was known. The required and actual sample sizes are listed in Table 1. Valid responses were obtained from one thousand respondents. After reaching the saturation point for the respective demographic segments, the sample ensured an approximately proportional representation of sex, age, and region categories.

**Table 1:** Determination of the respondent sample sizes

Variable	Stratum	Population size	Required sample size	Actual sample size	Margin of error (CL 90%)
Sex (SX)	Male (≥ 16 years)	2 194 165	271	470	3.79
	Female (≥ 16 years)	2 344 497	271	530	3.57
	Total (≥ 16 years)	4 538 663	271	1000	2.60
Age category (AC)	16–24	485 616	271	107	7.95
	25–39	1 167 420	271	280	4.91
	40–54	1 220 655	271	276	4.95
	> 55	1 644 788	271	337	4.48
Residence area	Bratislava (capital)	669 592	271	114	7.70
	Eastern Slovakia	1 627 704	271	338	4.47
	Central Slovakia	1 336 785	271	249	5.21
	Western Slovakia	1 823 792	271	299	4.76

The required sample sizes were calculated for a 5% margin and 90% confidence level (CL). The actual sample sizes corresponded to the number of completed and returned questionnaires

The survey questionnaire was developed on the basis of SRT and ART to explore and describe associations between SWB and preferences for forest features and activities, as well as to provide a set of ordinal predictors that would inform forest planning and public health policies. It was validated and distributed digitally to the above panel in cooperation with an established market research agency (Go4Insight) specializing in data collection and qualitative and quantitative research. It consisted of 10 binary and 7 Likert-scale questions used to capture and evaluate the respondents' agreement or disagreement with the proposed statements regarding the purpose of a forest visit and preference for forest features (Table 2). Data on perceived SWB and stress relief on the ordinal scale were taken from our earlier work (Pichlerová et al. 2023).

**Table 2:** Questionnaire statements regarding forest visitor activities and forest environment features

Area of perception	Item	Question or suggested statement	Possible response
Activity		What is the motive/purpose of your forest visits?	
	P1	Collecting fuel (coarse woody debris)	Did you make forest visits for the following purpose? 1 Yes 0 No
	P2	Picking mushrooms	
	P3	Picking forest berries	
	P4	Harvesting herbs for medicinal use	
	P5	Leisure walking	
	P6	Sports	
	P7	Hiking	
	P8	Barbecuing	
	P9	Camping	
	P10	Hunting	
Forrest patterns		Which forest feature is the most important for you?	To what extent do you agree with the following statements about forest visits? 1 Fully agree 2 Somewhat agree 3 Somewhat disagree 4 Fully disagree
	F1	Trees of different age	
	F2	Presence of old trees	
	F3	Trees of different species	
	F4	Trees of different height	
	F5	Presence of deadwood	
	F6	Forest smell	
	F7	Forest sounds	
Well-being and stress relief	SWB	After visiting the forest, I feel better than before	
	SR	After visiting the forest, I am less stressed and calmer	

Data on perceived subjective well-being (SWB) and stress relief (SR) on the ordinal scale were taken from our earlier works (Pichlerová et al. 2023). Forest uneven agedness was understood as stand diameter irregularity (Martín-Alcón et al. 2010). Hunting was partially exempt from the COVID-19 restrictions

### Statistical analyses

Statistical analyses on the data obtained from 1000 respondents were performed using the IBM SPSS Statistics version 28.0.1.0 (IBM Corp. Released 2021). A one-sample Wilcoxon signed-rank test was used to assess the deviation of the observed median from the hypothetical neutral value. Ward’s hierarchical clustering method with an agglomeration schedule was used to group forest activities. The clustering routine minimizes the pattern difference as a dissimilarity measure for binary data (Fonseca 2013). Clustering was computed as *bc*/*n*^2^, where *b* and *c* represent the diagonal cells corresponding to cases present on one activity but absent on the other; *n* is the total number of observations. Given a set of binary-valued independent variables (forest visit purposes), the probabilities of different outcomes of an ordinal variable (reported SWB improvement after forest visit) were analyzed using multinomial logistic regression. The resulting parameters with significant negative coefficients decrease, and those with positive coefficients increase the likelihood of that response category. The dependence of a polytomous response on a set of ordinal predictors was modeled by ordinal regression using the logit link function. The forest features important to the majority of respondents and representing distinct aspects of the forest environment were included in the model as explanatory variables. The main results of the ordinal regression analysis were estimates of the ordered log-odds (logit) regression coefficients. A unit change in the predictor would produce a change in the logit of the outcome by its respective parameter estimate, provided that the variables in the model are held constant (Mertens et al. 2017). Wald statistics and their corresponding *p*-values were used to test the null hypothesis. That is, the coefficient of the independent variable is equal to zero versus the alternative hypothesis, where the coefficient is nonzero (Forthofer et al. 2007). The ordinal model predictive capacity was expressed using Nagelkerke’s pseudo-*R*^2^ (Nagelkerke 1991). The results were considered statistically significant if *p* < 0.05 and marginally significant if 0.05 ≤ *p* < 0.10.

## Results and discussion

### Forest activities

**Fig. 1:**
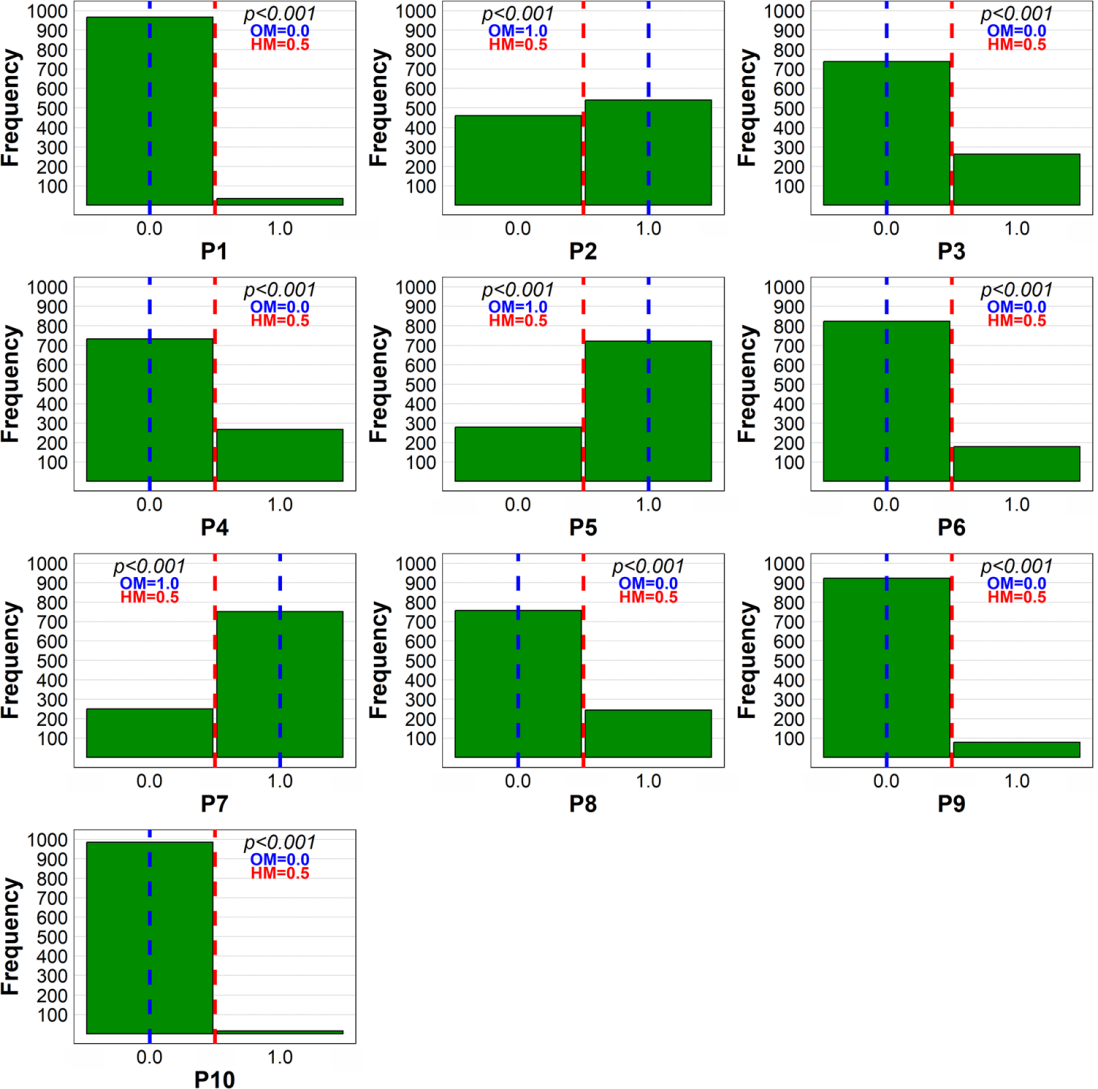
One-sample Wilcoxon Signed Rank Test of the differences between observed median (OM) values of the respondents’ answers regarding the purpose of their forest visits (1 = yes, 0 = no) and the hypothetical median (HM = 0.5). The dashed vertical lines indicate the hypothetical median (red) and observed median (blue) of the collected responses. *P1* collecting forest fuel, *P2* picking mushrooms, *P3* picking forest berries, *P4* harvesting herbs, *P5* leisure walking, *P6* sports, *P7* hiking, *P8* barbecuing, *P9* camping, *P10* hunting. The results are based on data from 1000 respondents

A substantial majority of the respondents engaged in recreational walking, hiking, and mushroom-picking in the given order (Fig. 1). The proportion of respondents who gave hunting as their forest visit purpose corresponded to the number of registered hunters in Slovakia (1.6% vs.1.2%, respectively), providing an ad hoc partial validation of the data reliability. The mushroom-picking was an important forest visit motive for nearly 50% of survey participants, paralleling Fennoscandian countries. For example, over half of Finns pick berries and mushrooms annually (Turtiainen et al. 2012).

**Fig. 2:**
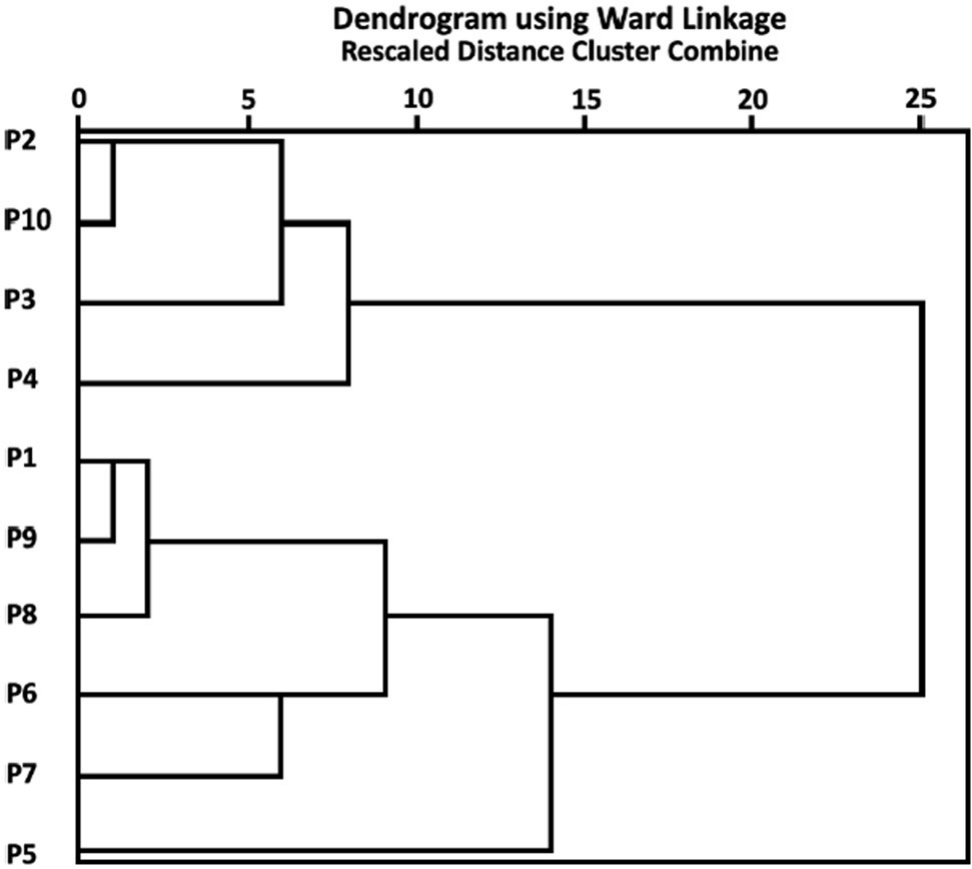
Hierarchical clusters of forest visit purposes: *P1* collecting fuel, *P2* mushroom-picking, *P3* berry-picking, *P4* collecting medicinal plants, *P5* recreational walk, *P6* sports, *P7* hiking, *P8* barbecuing, *P9* camping, *P10* hunting. The results are based on data from 1000 respondents

Hierarchical classification of stated forest visit activities based on pattern differences revealed clusters at various rescaled distances; clusters connected by lines (clads) further to the right are more dissimilar (Fig. 2). Successive clustering of related activities began with close relationships between the use of venison and mushrooms (P2 and P10), both popular in traditional game-based cuisine. This was followed by activities usually linked with the processing of berries and herbs for domestic consumption or sale in various forms, including fruit preserves, herbal teas, and others (P3, P4). Next was the use of forest fuel (P1) during camping (P9) and barbecuing (P8). At larger distances, the latter cluster was joined by sports (P6), hiking (P7), and leisure walking (P5). The emergence of two main clusters linked at the nearly maximum relative distance furthest from the right indicates a distinctive split between forest visit purposes aimed at provisioning (P2–4, P10) and recreation (P5–9). The only exception was the collection of forest fuel (P1) by barbecuers and campers.

Overall, large respondent segments specialized in either provisioning or recreational activities during their forest visits. These distinct affinities appear to be related to findings that people vary in their connectedness to nature manifested in recreational activities; some people interact with nature, while others may not consider such interactions an important part of their lives (Chang et al. 2020).

### Forest features

**Fig. 3:**
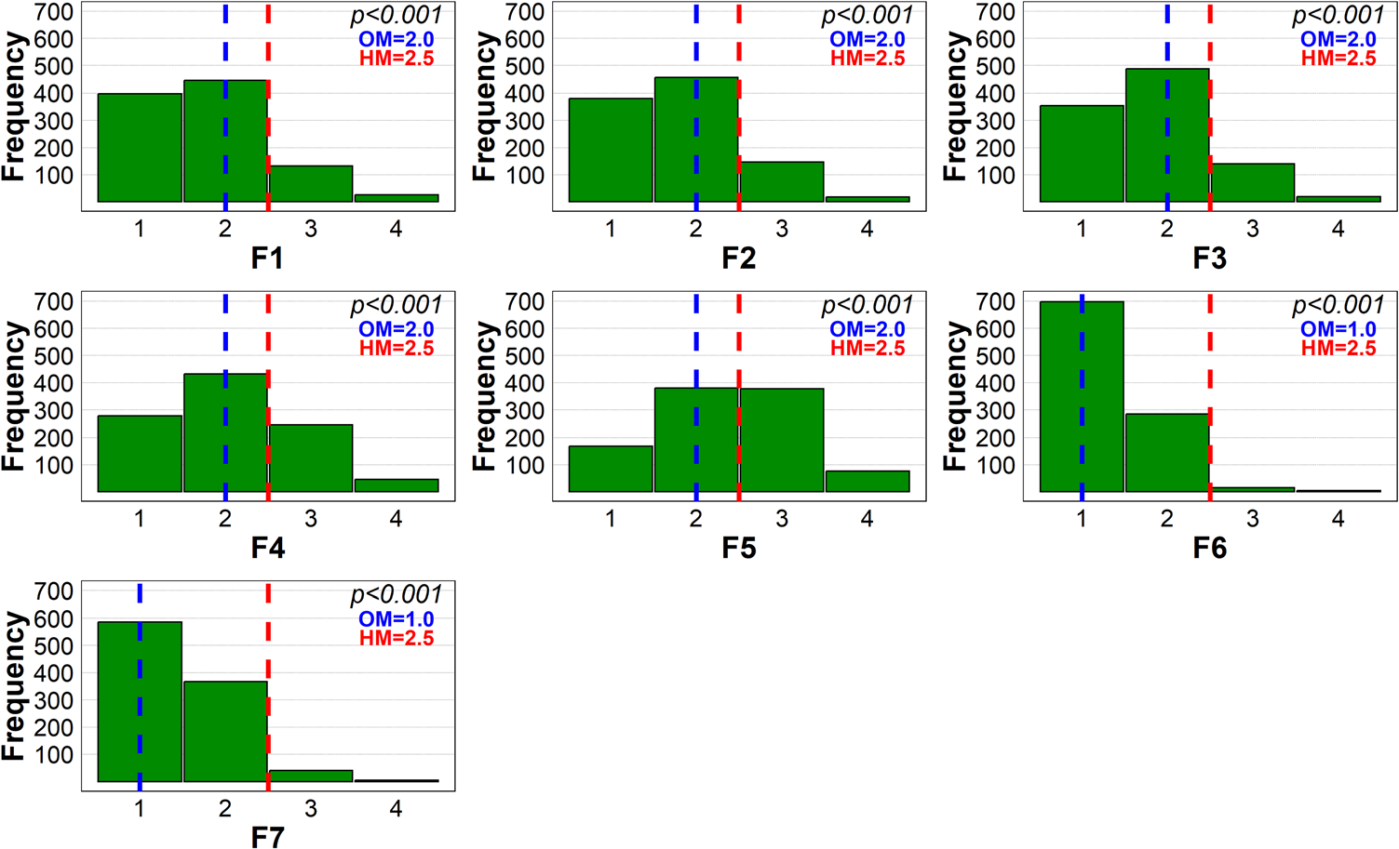
One-sample Wilcoxon Signed Rank Test of the differences between observed median (OM) values of the respondents’ answers regarding the most valued forest feature (1 = fully agree, 2 = somewhat agree, 3 = somewhat disagree, 4 = fully disagree) and the hypothetical median (HM = 2.5). The dashed vertical lines indicate the collected responses’ hypothetical median (red) and observed median (blue). *F1* trees of different ages, *F2* the presence of old trees, *F3* trees of different species, *F4* trees of different heights, *F5* the presence of deadwood, *F6* forest smell, *F7* forest sound. The results are based on data from 1000 respondents

A large majority of the respondents selected the presence of trees of different ages (F1), old trees (F2), different tree species (F3), forest sounds (F6), and forest smell (F7) as the most important forest characteristics (Fig. 3). In comparison, fewer people agreed with the importance of trees of different heights (F4) or the presence of deadwood (F5). Studies from Finland (Hauru et al. 2014) and Germany (Sacher et al. 2022) found that both old and fresh logs were rated higher in terms of aesthetic diversity than no deadwood. Moreover, deadwood enrichment did not conflict with the recreational value of forests. Stands without deadwood were preferred by respondents in Italy (Paletto et al. 2022) and Ukraine (Pelyukh et al. 2019). Our results show an intermediate position between the Scandinavian and West European perspectives and the South and East European perspectives on the deadwood. This could result from intense awareness-raising campaigns by state nature conservancies and non-governmental organizations. All observations were supported by the Wilcoxon test, and the results showed significant deviations in the sample median from the hypothetical neutral median value (2.5).

**Fig. 4:**
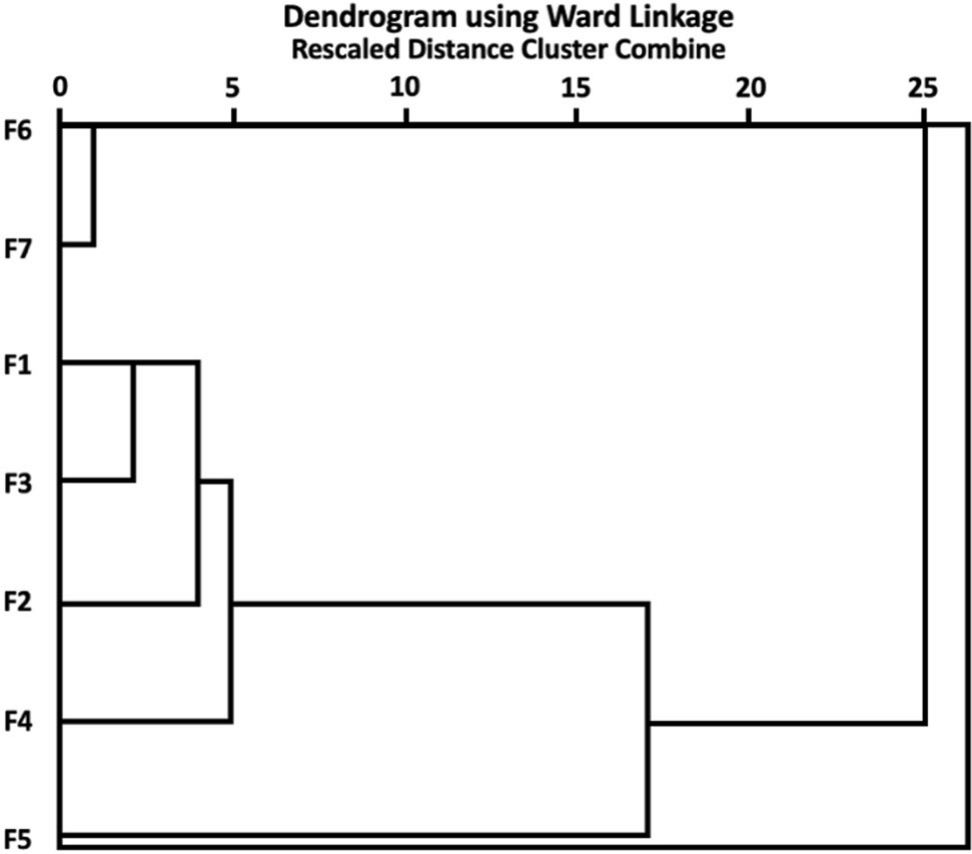
Hierarchical clustering of forest feature preferences: *F1* trees of different ages, *F2* the presence of old trees, *F3* trees of different species, *F4* trees of different heights, *F5* the presence of coarse woody debris (downed logs), *F6* forest smell, *F7* forest sounds. The results are based on data from 1000 respondents

Hierarchical classification of the preferences for forest characteristics produced two distant clusters comprising forest scent (F6) and sounds (F7), and visual impressions of forest structural diversity, comprising trees of different ages (F1), old trees (F2), different species (F3), different height and thickness (F4) (Fig. 4).

### SWB and stress reduction

#### SWB, stress reduction, and forest visit purpose

The division into provisioning and recreation warrants inquiry into which activities from the two clusters were strongly linked with increased SWB after the forest visit. This question was answered using multinomial logistic regression (Table 3). We established that feeling better after a forest visit showed significant (*p* = 0.038) and marginally significant (*p* = 0.056) positive associations with the preference for leisure walking and hiking, respectively. Different effects of various activities carried out in forests on anxiety and depression, cognitive function, stress, and other variables have recently been reported by Park et al. (2022). In our study, forest visit purpose explained approximately 12% of the variability in SWB. A more inclusive model comprising both natural- and outdoor-based activities explained 35% of the SWB variation (Beall et al. 2022).

**Table 3:** Multinomial logistic regression between strong agreement on feeling better after forest visit (Q1) and forest visit purposes (P1–P10)

Parameter estimates									
Variables		Estimate	Std. error	Wald	df	Sig	Exp(B)	95% confidence interval for Exp(B)	
								Lower bound	Upper bound
SWB = 1	Intercept	45.476	1850.888	0.001	1	0.980			
	P1 = 0	− 13.716	1514.253	0.000	1	0.993	1.105E–6	0.000	0.^b^
	P1 = 1	0^a^			0				
	P2 = 0	0.163	0.663	0.061	1	0.806	1.177	0.321	4.320
	P2 = 1	0^a^			0				
	P3 = 0	− 0.343	0.846	0.165	1	0.685	0.709	0.135	3.724
	P3 = 1	0^a^			0				
	P4 = 0	− 0.739	0.840	0.774	1	0.379	0.477	0.092	2.478
	P4 = 1	0^a^			0				
	P5 = 0	− 1.307	0.629	4.320	1	0.038	0.271	0.079	0.928
	P5 = 1	0^a^			0				
	P6 = 0	0.366	0.807	0.206	1	0.650	1.442	0.296	7.012
	P6 = 1	0^a^			0				
	P7 = 0	− 1.207	0.630	3.664	1	0.056	0.299	0.087	1.029
	P7 = 1	0^a^			0				
	P8 = 0	0.308	0.829	0.138	1	0.711	1.360	0.268	6.907
	P8 = 1	0^a^			0				
	P9 = 0	− 13.643	1064.342	0.000	1	0.990	1.189E–6	0.000	0.^b^
	P9 = 1	0^a^			0				
	P10 = 0	− 13.260	0.637	433.708	1	< .001	1.742E–6	5.001E–7	6.068E–6
	P10 = 1	0^a^			0				
SWB = 2 SWB = 3 SWB = 4^a^	No significant values of parameter B estimates for these factor levels								

Link function: Logit; *p* < 0.001; Nagelkerke's pseudo-*R*^2^: 0.121

Abbreviations and explanatory notes: *Q1* Fully agree that after visiting the forest, I feel better than before, *P1* collecting forest fuel, *P2* picking mushrooms, *P3* picking forest berries, *P4* herbs harvesting, *P5* leisure walking, *P6* sports, *P7* hiking, *P8* barbecuing, *P9* camping, *P10* hunting. The reference category is SWB = 4—I don’t agree that I feel better after visiting the forest than before. There were no significant parameters for the intermediate responses SWB = 2, 3, and the corresponding rows are therefore not shown

^a^This parameter is set to zero because it is a reference value

^b^Floating point overflow occurred while computing this statistic and its value is therefore set to system missing. The results are based on data from 1000 respondents

Furthermore, a significant link (*p* < 0.001) was observed between SWB and hunting. This is noteworthy, as people working in forestry perceive natural environments as less restorative than others (von Lindern et al. 2013). We hypothesize that hunting can entail jouissance—enjoyment located beyond the pleasure principle (Lacan 2021). This is generated due to the paradox of a hunter killing the animals they purport to love (Andersson Cederholm and Sjöholm 2021; Cohen 2014). Such an experience happens concurrently with reflecting, moving, and exploring the human mind and body with the forest world. Häggström (2019a, 2019b) conceptualized such a world, including animals, as “more-than-human” and pointed out the deeply anchored human connection to it. This connection could grow even stronger owing to a partial loss of control over one’s life during the pandemic. No other provisioning activity was associated with improved SWB, indicating that such activities were perceived as practical and possibly even tiring rather than reinvigorating.

#### SWB, stress reduction, and forest features

When included in an ordinal regression model, the preferences for forest patterns explained approximately 15% of the variability in SWB (Table 4). Our recollection-derived data analysis showed a significant positive association between SWB and preference for uneven-aged forests (F1, *p* = 0.049). In contrast, SWB improvement decreased with increasing preference for a higher degree of structural diversity produced by the variable tree height (F4) (*p* < 0.013). It is apparent that people feel less comfortable in forests that cannot be easily monitored visually for possible risks. For example, a study on images of dense hardwood forest, rainforest, or prairie indicated that preferences were low for areas that were visually blocked or lacking spatial definition, where one’s ability to anticipate potential dangers was constrained by dense foliage or high grass (Kaplan and Kaplan 1989). However, the latter authors also indicated that people’s expression of satisfaction might not be directly linked to the actual use of a natural setting. We posit that there are relevant differences between results derived from recollections of individual forest visits, such as in this study, experiments in controlled settings, and viewing images. For instance, Filyushkina et al. (2017) reported that respondents who could select stands from a catalogue of drawings preferred mixed, uneven-aged forests, and stands with variable tree height. Ebenberger and Arnberger (2019) found that four different tree heights was the most preferred attribute when presented on digital forest images, and an old-growth forest featuring high structural diversity and a mature commercial forest emerged as more restorative than an urban forest or a young commercial stand during a supervised field experiment (Simkin et al. 2020). Thus, people who make individual forest visits might prefer forests with high structural diversity (F4), they could also avoid them because of the potential risks stemming from unexpected encounters with a brown bear whose population in the Slovak forests is relatively high (Skuban et al. 2017). Recent findings by Beute and de Kort (2018) indicate that individual preferences for the natural environments could remain relatively unaffected by negative associations with nature.

**Table 4:** Ordinal regression between the subjective well-being after forest visit (SWB) and preferences for forest features typically experienced in the forest environment, including sensory stimuli

Parameter estimates							
Variables	Estimate	Std. error	Wald	df	Sig	95% confidence interval	
						Lower bound	Upper bound
Threshold							
SWB = 1.00	− 0.758	1.081	0.492	1	0.483	− 2.877	1.360
SWB = 2.00	2.149	1.086	3.917	1	0.048	0.021	4.278
SWB = 3.00	3.867	1.119	11.938	1	< .001	1.674	6.061
Location							
F1 = 1.00	− 1.023	0.519	3.890	1	0.049	− 2.039	− 0.006
F1 = 2.00	− 0.880	0.508	3.004	1	0.083	− 1.876	0.115
F1 = 3.00	− 0.890	0.524	2.887	1	0.089	− 1.917	0.137
F1 = 4.00	0^a^			0			
F4 = 1.00	1.158	0.465	6.201	1	0.013	0.247	2.069
F4 = 2.00	1.479	0.443	11.167	1	< .001	0.612	2.347
F4 = 3.00	1.482	0.443	11.190	1	< .001	0.614	2.351
F4 = 4.00	0^a^			0			
F6 = 1.00	− 0.582	1.081	0.289	1	0.591	− 2.701	1.537
F6 = 2.00	0.317	1.083	0.086	1	0.770	− 1.805	2.440
F6 = 3.00	0.356	1.205	0.088	1	0.767	− 2.005	2.717
F6 = 4.00	0^a^			0			
F7 = 1.00	− 1.443	0.966	2.230	1	0.135	− 3.338	0.451
F7 = 2.00	− 0.649	0.965	0.453	1	0.501	− 2.540	1.241
F7 = 3.00	− 0.247	1.009	0.060	1	0.806	− 2.225	1.730
F7 = 4.00	0^a^			0			

Link function: Logit; *P* < 0.001; Nagelkerke's pseudo-*R*^2^: 0.148

Abbreviations and explanatory notes: *F1* trees of different ages, *F4* trees of different heights, *F6* forest smell, *F7* forest sound. In SWB, F1, F4, and F7, the indexes 1–4 correspond to statements “Fully agree,” “Somewhat agree,” “Somewhat disagree,” and “Fully disagree” with the importance of a given forest feature, respectively

^a^ This parameter is set to zero because it is a reference value. The results are based on data from 1000 respondents

The effects of preference for selected forest features on stress reduction were partly different (Table 5). While there was a marginally significant association (*p* < 0.1) with a preference for trees of variable age (F1), a much stronger positive association emerged between stress reduction and forest smell (*p* < 0.001). Nature and forests in this study are abundant in smells from trees, shrubs, flowers, rotting matter (such as dead wood), and other sources (Franco et al. 2017). Interestingly, our recollection-based findings correspond to recent results from controlled laboratory experiments reported by Hedblom et al. (2019). They established that forest smell was the only factor that predicted both self-reported and physiologically determined stress levels. In contrast to the position of olfactory receptors—only two synapses away from the amygdala and hypothalamus (the two key nodes in initial stress responses)—all other sensory systems connect to cerebral areas via multi-synaptic pathways (Gray 1993; Hedblom et al. 2019). Our results support the hypothesis that while individual contributions of multiple pathways between nature and health may be small, their cumulative effect could be substantial (Kuo 2015).

**Table 5:** Ordinal logit regression between the perceived stress relief (SR) after forest visit and forest features, including sensory stimuli typically experienced in the forest environment

Parameter estimates							
Variables	Estimate	Std. error	Wald	df	Sig	95% confidence interval	
						Lower bound	Upper bound
Threshold							
SWB = 1.00	− 1.941	1.128	2.960	1	0.085	− 4.151	0.270
SWB = 2.00	1.109	1.124	0.974	1	0.324	− 1.094	3.313
SWB = 3.00	2.805	1.142	6.029	1	0.014	0.566	5.044
Location							
F1 = 1.00	− 0.869	0.500	3.017	1	0.082	− 1.850	0.112
F1 = 2.00	− 0.695	0.490	2.011	1	0.156	− 1.655	0.265
F1 = 3.00	− 0.697	0.509	1.877	1	0.171	− 1.694	0.300
F1 = 4.00	0^a^			0			
F4 = 1.00	0.515	0.405	1.611	1	0.204	− 0.280	1.309
F4 = 2.00	0.724	0.383	3.565	1	0.059	− 0.028	1.475
F4 = 3.00	0.468	0.387	1.465	1	0.226	− 0.290	1.226
F4 = 4.00	0^a^			0			
F6 = 1.00	− 3.801	1.095	12.044	1	< .001	− 5.948	− 1.655
F6 = 2.00	− 2.891	1.093	6.991	1	0.008	− 5.033	− 0.748
F6 = 3.00	− 2.332	1.212	3.704	1	0.054	− 4.707	0.043
F6 = 4.00	0^a^			0			
F7 = 1.00	1.374	1.144	1.444	1	0.230	− 0.867	3.616
F7 = 2.00	2.291	1.143	4.017	1	0.045	0.051	4.532
F7 = 3.00	2.253	1.182	3.631	1	0.057	− 0.064	4.571
F7 = 4.00	0^a^			0			

Link function: Logit; *p* < 0.001; Nagelkerke's pseudo-*R*^2^: 0.197

Abbreviations and explanatory notes: *F1* trees of different ages, *F4* trees of different heights, *F6* forest smell, *F7* forest sound. In SWB, F1, F4, and F7, the indexes 1–4 correspond to “Fully agree,” “Somewhat agree,” “Somewhat disagree,” and “Fully disagree” with the suggested statements, respectively

^a^ This parameter is set to zero because it is a reference value. The results are based on data from 1000 respondents

The results concerning the preference for auditory stimuli (forest sounds) are mixed. There were non-significant tendencies towards SWB improvement but a lack of stress reduction after forest visits, with an increasing preference for forest sounds. Even controlled multisensory virtual reality experiments have produced only a statistical tendency in the association between auditory stimuli and lower stress responses (Hedblom et al. 2019). Ratcliffe et al. (2013) showed that bird sounds contribute to perceived attention restoration and stress recovery as a part of natural soundscapes. However, our study defined forest sounds more broadly to include branch cracking or rubbing against each other, and animal sounds. From an evolutionary perspective, studies have examined humans alertness to patterns that signal danger and security (Buxton et al. 2021; Katcher and Wilkins 1993). Therefore, tree branches breaking or hitting the ground, animal sounds, or even deep silence could be perceived as danger signals or increased alertness, thus preventing stress reduction (e.g., in hunters).

### Study limitations

Our study has several limitations. All analyses were based on a questionnaire survey and relied on people’s recollections. Therefore, we assumed that people’s cognitive evaluations, decisions, and acts were memory- or recollection-based (Gigerenzer and Selten 2001; Khader et al. 2011). Specifically, imperfect recall and knowledge imperfection play a paramount role in the human processing of information (Rubinstein 1998). In addition, the survey omitted positive emotion generation and stress reduction during nature and forest recreation in dyads or groups, as conceptualized through relational restoration theory that emphasizes supportive exchanges between people (Hartig 2021). However, because pandemic-related restrictions in Slovakia and many other countries did not permit natural and forest visits by mixed groups at that time, we assumed that most people visited forests as individuals or families. Other relevant factors not accounted for in this study were childhood forest experiences, forest-related personal and cultural identities (Häggström, 2019a), and place attachment (Korpela et al. 2009). Finally, the questionnaire did not cover forest visit duration. The impact of missing information can be mitigated by the lack of evidence that longer time spent on happier activities leads to higher levels of reported well-being (Henwood et al. 2022).

### Recommendations for further research and place management

The recollection-based results agreed with recent laboratory findings regarding the importance of forest smell for stress relief. At the same time, they reflect forest visitors’ awareness of the potential risks and dangers typical of the real forest environment, which are usually not regarded under controlled settings. Emotions and responses triggered by them, such as fear and anxiety, usually affect information processing and help to select particular aspects of the stimulus environment (Adolphs and Damasio 2001). For instance, the associations between SWB or stress reduction and preference for forest sounds are ambiguous. Hence, further studies must be also conducted in real-world forests, account for usual forest visitor routines, and consider climate change-induced phenomena, such as rising ambiental temperature and an increased occurrence of sudden weather changes. E.g., Ebenberger and Arnberger (2019) found indications that seeking relief from temperatures rising due to climate change is already an important reason for forest visits, even in localities with lesser aesthetic appeal. Also, the effect of forest visits on SWB is increasingly likely to be conditioned on the availability of convenient networks of shelters, which are used by forest visitors in case of sudden weather changes, mainly storms (Kotásková et al. 2018). Additional research should also account for eco-anxiety and other human–environment domains that can affect forest restoration, e.g., the presence or absence of the feelings of freedom or gratitude as important positive emotions engendered by forest visits (Pichlerová et al. 2023). Such studies may inform practical forest management on establishing restorative visual scenery, soundscapes, and smellscapes, especially in urban and other forests with recreational and restorative functions (Yamada 2006). Because the preferences for forest features explained a comparable or larger portion of the SWB variability, they should be considered on par with forest activities; they cannot be omitted in designing and maintaining restorative forest environments. In addition, the link between SWB and stress reduction during and after time spent in forests by hunters and non-hunters warrants further research. Although hunting represents a comparatively small part of forest activities, it may engender mixed responses from other forest visitors (Reis and Higham 2009).

## Conclusions

The presented study revealed specific associations between subjective well-being (SWB) and stress reduction after forest visits on the one hand, and the preferences for certain forest features and forest-derived sensory stimuli, mainly forest uneven agedness and forest smell on the other hand. Up to 20% and 12% of SWB increase and stress reduction variability were explained by these forest features and mainly recreational forest activities, respectively. The results align with the first hypothesis that SWB and stress reduction after forest visits are associated with preferences for certain forest features and activities. The second hypothesis that provisioning forest activities would better predict SWB than recreational activities was not confirmed despite the former’s tangible material benefits. The findings also show that the recollection-based results obtained from exposure to real-world forest settings may be affected by biophobia, which is usually not felt in virtual or controlled forest environments. As the recollections-based results integrate an array of forest environmental factors and people’s responses to them, they can be generalized and used to conserve, design, or manage forests that provide robust mental health benefits to forest visitors.

## Data Availability

The datasets generated during and/or analysed during the current study are not publicly available due to legal reasons but are available from the corresponding author on reasonable request.
